# Giant descending aortic pseudo-aneurysm in an adult man with uncorrected aortic coarctation

**DOI:** 10.1186/s43044-021-00206-0

**Published:** 2021-09-16

**Authors:** Zahra Jabbary, Mehrnoush Toufan

**Affiliations:** Cardiovascular Research Center, Shahid Madani Heart Center, Tabriz University of Medical Sciences, Tabriz, Iran

**Keywords:** Aortic pseudo-aneurysm, Aortic coarctation, Echocardiography

## Abstract

**Background:**

Aortic coarctation (CoAo) accounts for 6 to 8% of all congenital heart diseases and occurs two to five times more often in males. The uncorrected aortic coarctation is complicated by hypertension, ascending and descending aortic aneurysms, endarteritis, and heart failure. The aortic pseudo-aneurysm (APD) usually occurs in patients with endarteritis. We report an adult man with bicuspid aortic valve, perimembranous ventricular septal defect, and uncorrected aortic coarctation complicated by descending aortic pseudo-aneurysm without aortic endarteritis.

**Case presentation:**

A 40-year-old man was referred to our division for hemoptysis and severe aortic coarctation. Echocardiography confirmed the aortic coarctation diagnosis and showed a large aortic pseudo-aneurysm at the coarctation site with intra-cavity mural thrombus. Subsequently, the patient underwent contrast-enhanced computed tomography angiography, and diagnosis of coarctation and APD was confirmed. Due to various malformations and considering that the patient had become unstable due to hemoptysis, it was discussed in the heart team, and it was decided that the patient would undergo staged surgery.

**Conclusions:**

The aortic pseudo-aneurysm is a rare complication in patients with untreated coarctation that requires prompt surgery, and this complication should be considered in patients with untreated aortic coarctation who present with hemoptysis.

## Background

Aortic coarctation (CoAo) accounts for 6–8% of all congenital heart diseases and affects 4/10,000 live births with male dominance. The coarctation has a wide anatomical variation, it may appear as an isolated stricture or long-segment arterial narrowing. Regardless of its anatomical morphology, all individuals with aortic coarctation present a diffuse arteriopathy. The accompanying anatomical abnormalities include bicuspid aortic valve (50–60%), subaortic membrane, mitral valve abnormalities, patent ductus arteriosus, ventricular septal defect, and aortic arch anomalies [1]. The untreated aortic coarctation is complicated by hypertension, ascending and descending aortic aneurysms, endarteritis, and heart failure [2]. The aortic pseudo-aneurysm (APD) usually occurs in patients with endarteritis [3]. We report an adult man with bicuspid aortic valve (BAV), perimembranous ventricular septal defect (VSD), and uncorrected aortic coarctation complicated by descending aortic pseudo-aneurysm without aortic endarteritis.

## Case presentation

A 40-year-old man with hemoptysis, and aortic coarctation was referred to our hospital. His medical history included smoking, hypertension, and uncorrected aortic coarctation. On clinical examination, a holodiastolic murmur was heard along the left margin of the sternum in the fourth intercostal space. The femoral pulses were weak, and a radial-femoral delay was observed. Blood pressure in the right and left arms was 140/80 mm Hg and 160/80 mm Hg, respectively. There was a systolic pressure gradient of about 60 mm Hg between the upper and lower extremities.

**Fig. 1:**
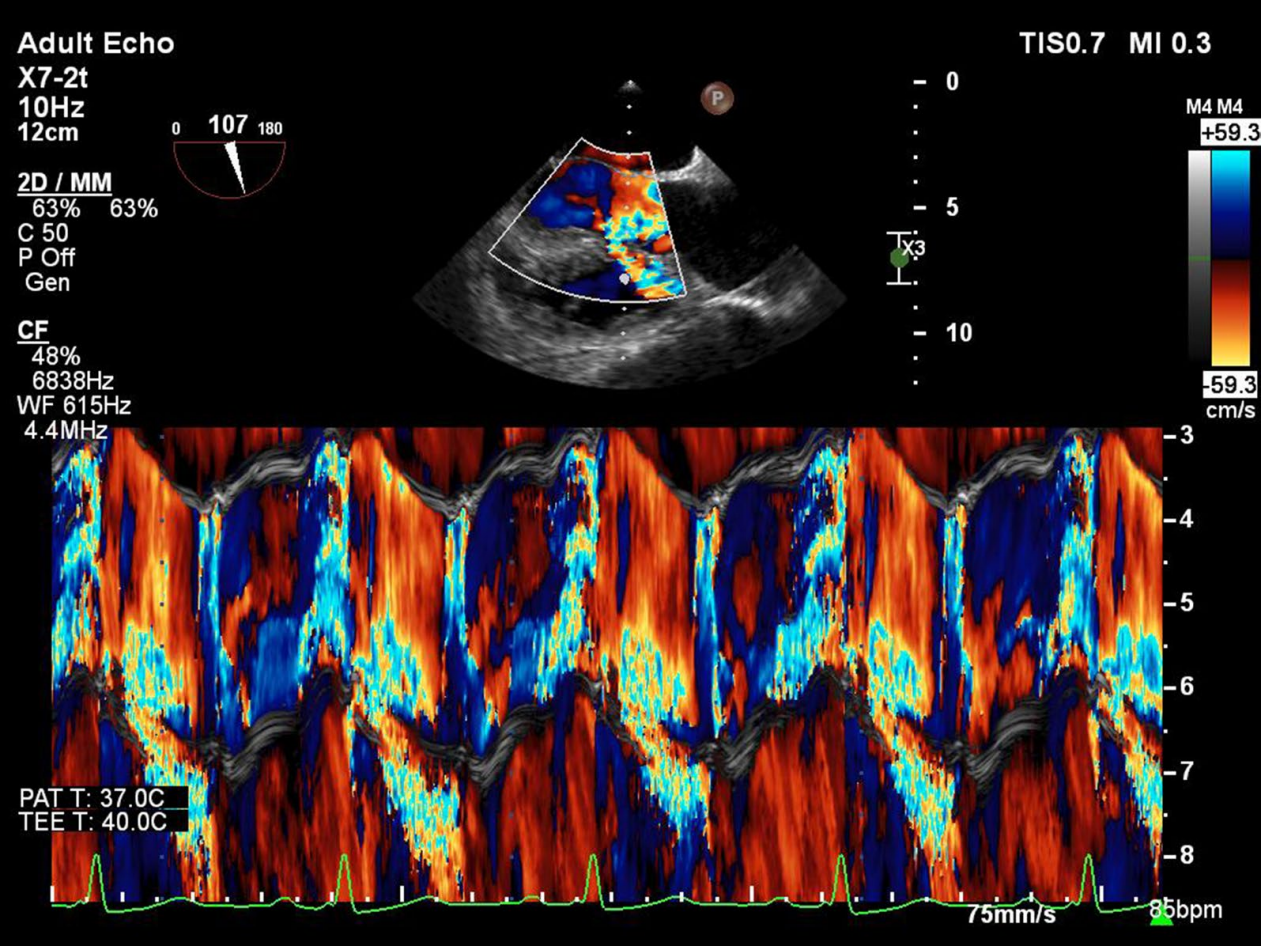
2D-TEE, Color-coded M-mode showing the aortic insufficiency (AI) signal during diastole, and the perimembranous VSD signal during systole

**Fig. 2:**
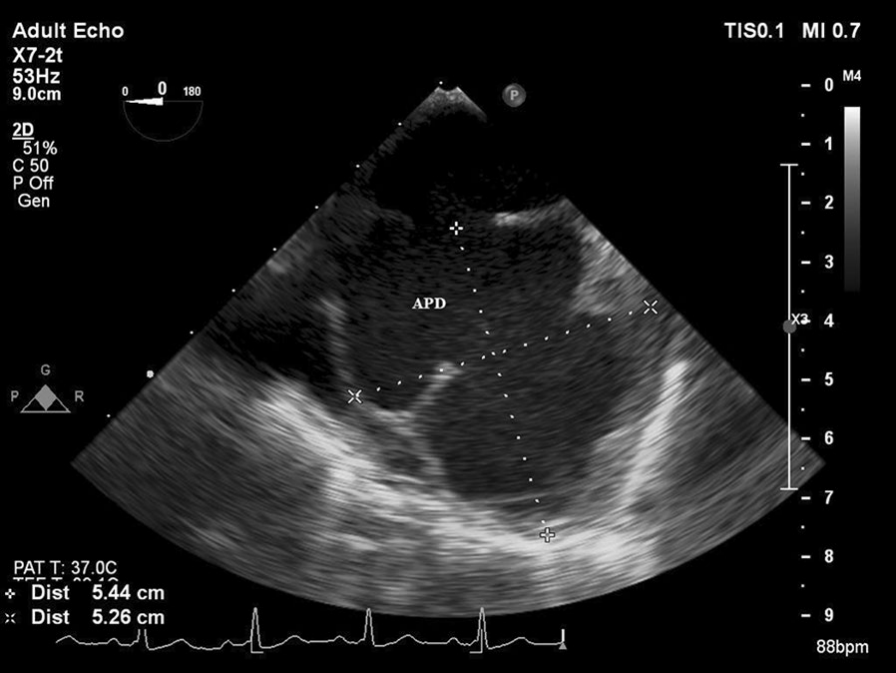
2D-TEE shows a large pseudo-aneurysm at the coarctation site with intra-cavity mural thrombus (*APD* aortic pseudo-aneurysm)

**Fig. 3:**
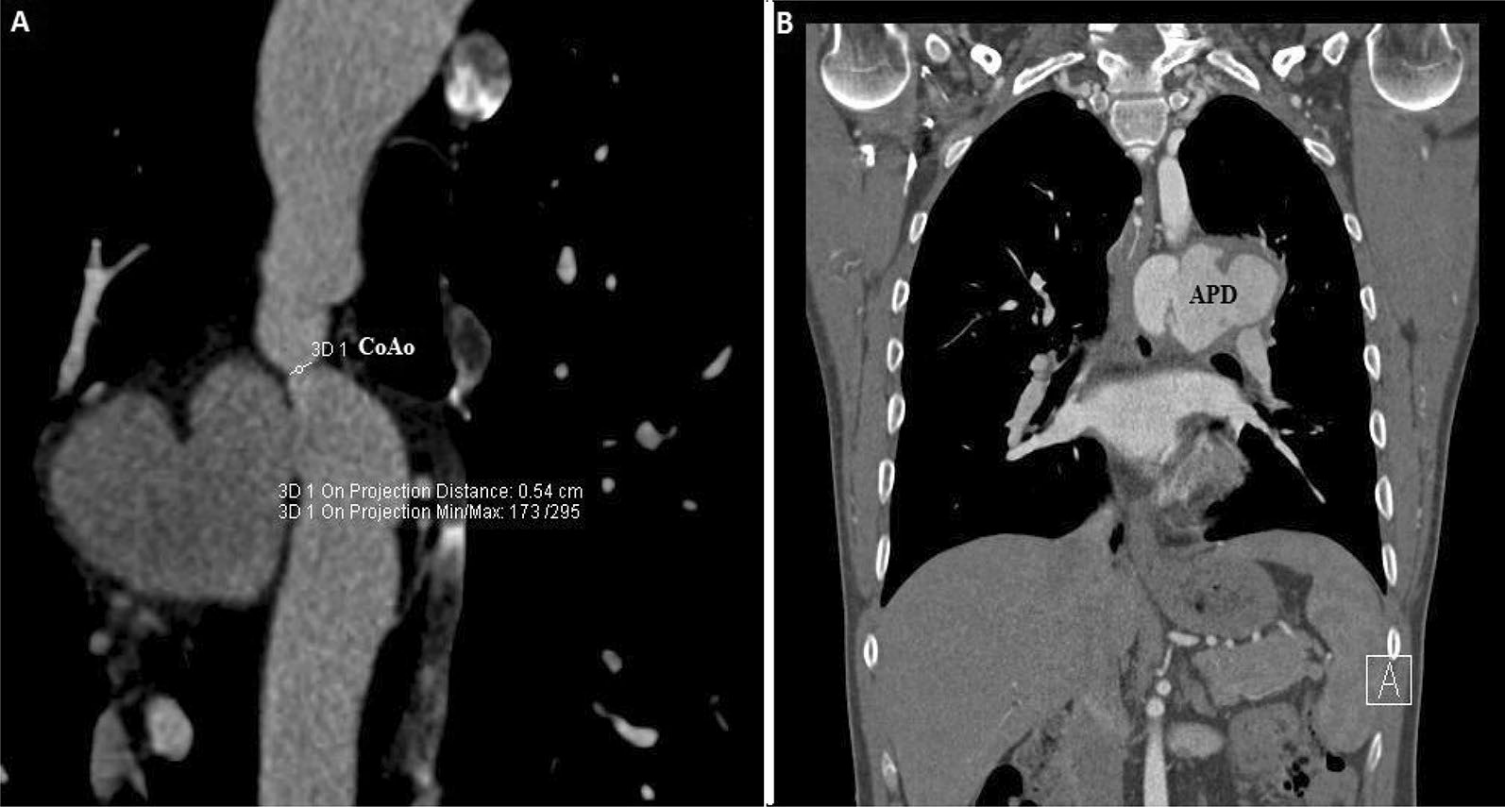
2D Computed tomography angiography scan shows a severe aortic coarctation and a giant pseudo-aneurysm at coarctation site (*CoAo* aortic coarctation, *APD* aortic pseudo-aneurysm)

**Fig. 4:**
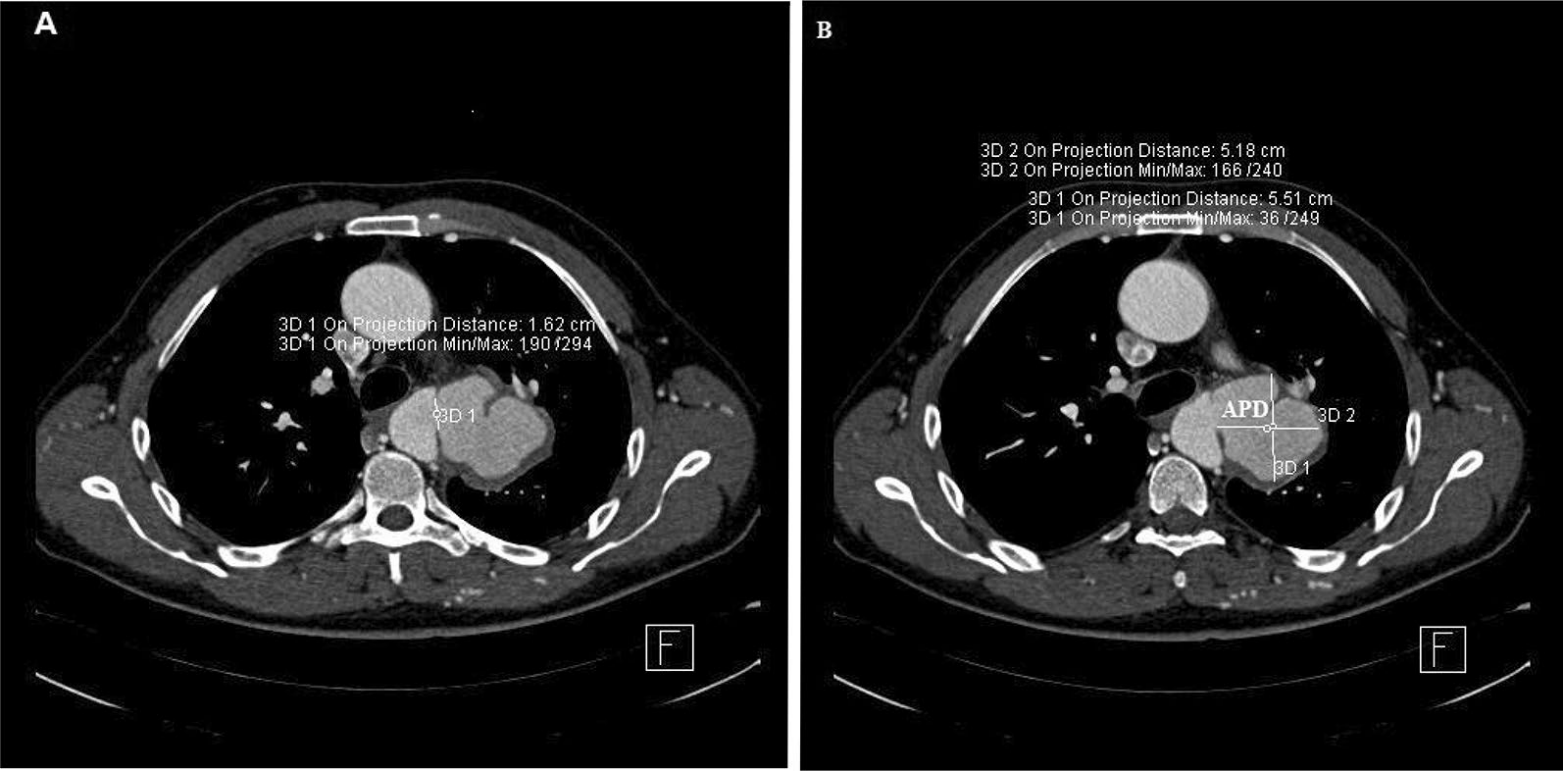
2D computed tomography angiography scan, axial views, shows the 52 × 55 mm pseudo-aneurysm with a small neck communicated to the aortic lumen (*APD* aortic pseudo-aneurysm)

First, two-dimensional transthoracic echocardiography (2D-TTE) was performed and showed a restrictive perimembranous ventricular septal defect (VSD) with peak gradient 119 mmHg, bicuspid aortic valve with severe insufficiency, and dilatation of the ascending aorta (approximately 46 mm) associated with a typical aortic coarctation at typical site (Fig. 1). There was maximal gradient of 70 mm Hg on Doppler study. Subsequently, the patient underwent two-dimensional transesophageal echocardiography (2D-TEE) in the presence of a cardiac anesthesiologist. In order not to put the patient at risk, we used adequate sedation during the two-dimensional transesophageal echocardiography. In addition to the above findings, a large pseudo-aneurysm was observed at the coarctation site with intra-cavity mural thrombus. The aortic pseudo-aneurysm (APD) measured 54.4 mm × 52.6 mm and with a small neck communicated to the aortic lumen (Fig. 2). Contrast-enhanced computed tomography angiography confirmed the diagnosis of coarctation and APD, and there was no evidence of contrast leakage from the aorta (Figs. 3, 4).

Due to various malformations and considering that the patient had become unstable due to hemoptysis, it was discussed in the heart team, and it was decided that the patient would undergo staged surgery. In the first stage, the left common carotid artery was bypassed by means of an 8-mm-Dacron graft to the left subclavian artery. After bypass, a 24–24–130-mm-JOTEC stent graft was deployed at the site of aortic coarctation and APD. The second stage of surgery, which is related to the correction of aortic insufficiency and VSD, has not been performed yet.

## Discussion

Coarctation occurs two to five times more often in males and has a high degree of association with gonadal dysgenesis (Turner syndrome) and bicuspid aortic valve (≥ 50%). Other common abnormalities associated with coarctation include VSD and mitral stenosis or regurgitation. Additional abnormalities have an impact on the outcome. In our patient, coarctation was associated with VSD and BAV [4].

Congenital heart diseases are rare causes of hemoptysis. Adult patients with congenital heart diseases such as aortic coarctation may rarely, present with hemoptysis as the only clinical sign due to high pressure and dilation in the bronchial arteries.

Hemoptysis may occur several years after the intervention due to aortic aneurysm and aortobronchial fistula, which is associated with a high risk of mortality (25–41%). In aortic coarctation, mechanical vascular obstruction leads to the development of bronchopulmonary collaterals with bronchial and systemic arterial hypertrophy [5]. High pressure with weakening of the bronchial vessel wall leads to hemoptysis and that was the reason of our patient’s hemoptysis. The patient did not report hemoptysis after surgery.

90% of patients with uncorrected aortic coarctation die up to the age of 50**.** Therefore, with timely intervention, the survival of these patients can be improved.

Death in patients with unrepaired aortic coarctation often occurs due to heart failure, aortic rupture or dissection, coronary artery disease, concomitant aortic valve disease, infective endarteritis or endocarditis, or cerebral hemorrhage [4]. The 25% of cause for death from untreated aortic coarctation is an acute rupture of the aorta or dissected dilated aorta [6]. Therefore, correction of APD before rupture is necessary in these patients. The cause of APD is not known, however, the possible causes suggested for the formation of APD include hypertension in the upper part of the body, congenital weakness in the aortic wall, jet stream through the coarctation, and infection [7]. Given that in our patient, APD was on the low blood pressure side of coarctation, so hypertension could not be the cause of pseudo-aneurysm formation. However, the possible causes of APD formation in our patient can be jet stream and congenital weakness of the aortic wall.

The gold standard for diagnosing APD is angiography; however, diagnosis is often made by transesophageal echocardiography, computed tomography with 3-dimensional reconstruction, and magnetic resonance angiography [8, 9]. The APD in our patient was identified by echocardiography, and contrast-enhanced computed tomography angiography confirmed the diagnosis.

The surgical procedure and time of surgical intervention are still controversial [10]. End-to-end anastomosis, patch formation, subclavian flap aortoplasty, and artificial blood vessel replacement are surgical methods that are used. Although the main method of treatment in children is surgery. In adults, endovascular treatment of coarctation with balloon angioplasty and stenting is used widely [11]. Our patient was referred for cardiothoracic surgery after the heart team decided on a treatment plan.

## Conclusions

The aortic pseudo-aneurysm is a rare complication in patients with untreated coarctation that requires prompt surgery, and this complication should be considered in patients with untreated aortic coarctation who present with hemoptysis.

## Data Availability

Not applicable.

## Abbreviations

CoAo: Aortic coarctation
APD: Aortic pseudo-aneurysm
BAV: Bicuspid aortic valve
VSD: Ventricular septal defect
2D-TTE: Two-dimensional transthoracic echocardiography
2D-TEE: Two-dimensional transesophageal echocardiography
AI: Aortic insufficiency
